# In vitro evaluation of simulated stereotactic radiotherapy for wet age-related macular degeneration on three different cell lines

**DOI:** 10.1038/s41598-021-87466-7

**Published:** 2021-04-13

**Authors:** Efstathios Vounotrypidis, Anna Hillenmayer, Christian M. Wertheimer, Alexis Athanasiou, Jakob Siedlecki, Michael Orth, Andreas Ohlmann, Siegfried G. Priglinger, Armin Wolf

**Affiliations:** 1 Department of Ophthalmology, University Hospital Ulm, Prittwitzstrasse 43, 89075 Ulm, Germany; 2 Department of Ophthalmology, Ludwig-Maximilians-University Munich, Munich, Germany; 3 Department of Radiation Oncology, University Hospital, LMU Munich, Munich, Germany

**Keywords:** Diseases, Medical research

## Abstract

Low energy stereotactic radiotherapy has been proposed for the treatment of neovascular age related macular degeneration. We investigated the in vitro effect of the radiotherapy on pericytes, retinal pigment epithelium and endothelial cells. Primary human retinal pigment epithelium cells, human umbilical vein endothelial cells and human pericytes from Placenta were cultivated. In a pairwise protocol, one plate was irradiated at a dose of 16 Gy, while the second plate served as a non-irradiated control. Thereafter, cells were cultivated either in serum-free (non-permissive) or serum-stimulated (permissive) conditions. A life/dead assay, an XTT and a BrdU assay were performed up to 7 days after irradiation. No cell death occurred at any timepoint in any cell line after treatment nor in the control. Compared to the unirradiated controls, cell viability and metabolic activity were significantly reduced in irradiated cells in the XTT assay, except for non-permissive RPE cells. In the BrdU assay, proliferation was inhibited. While no cell death was detected in vitro, viability and proliferative capacity of all cell lines were significantly reduced. Therefore, it seems that low energy stereotactic radiotherapy inhibits angiogenesis without a direct induction of apoptosis but influencing microvascular function and stability.

## Introduction

Intravitreal injection of vascular endothelial growth factor antibodies (anti-VEGF) revolutionized the therapy of neovascular AMD (nAMD)^1,2^. However, due to their necessary repeated application, they are associated with a high treatment burden^3^. The search for alternative therapies led to the investigation of stereotactic radiotherapy (SRT) using low-dose X-ray irradiation on the macular region of nAMD patients^4,5^. Through reduction of the needed anti-VEGF injection number, irradiation would, for one, lower the treatment cost of nAMD^6^. Hypothetically, possible morphological benefits include the reduction of an inflammatory response^7^, the inhibition of fibroblasts and therefore the reduction of scar formation^8^ and the death of rapidly dividing endothelial cells^9^. These morphological results were confirmed by clinical study findings, which suggested a possible additive effect of X-ray irradiation in addition to anti-VEGF, leading to a reduced number of injections^10–15^. Yet even though the injection frequency was reduced, visual acuity did not statistically improve compared to the injection only group and morphological long-term effects remain mainly undetermined^16^.

Radiation retinopathy—a result of microvascular damage through radiotherapy- is known to occur many years after the exposure^17,18^. Although describing studies report radiation retinopathy as a consequence of ocular tumor treatment, similar morphological changes were also observed in adjuvant stereotactic radiotherapy in nAMD treatment after a follow-up period of up to 3 years^19^. During the study the occurrence of microvascular anomalies increased in a time dependent manner and a significant visual loss was observed in comparison to monotherapy with anti-VEGF^16,19^. It has to be taken into account however, that anti-VEGF was administered as the main therapy in addition to the SRT, which mitigates the development of radiation retinopathy and visual loss^20^. The potential of reducing the burden of high injection frequencies in nAMD-patients is of major clinical interest, but might be halted by the side effects of SRT occuring late after the treatment and then reduce the treatment success.

To explain this effect, several studies previously examined the effect and morphological changes of low-dose irradiation in vitro on porcine and human retinal pigment epithelium (RPE) cells^21–23^, human endothelial cells^24–27^ and rat retinal cell cultures^28^. In conclusion, irradiation induced an antiproliferative effect on RPE cells^21–23^ and led to senescence, increased apoptotic rate, increased stiffness and dysfunctionality of human umbilical vein endothelial cells (HUVEC)^24–27^. Recently, another study showed a pericyte (hPC) loss in necrotic human brain tissue after irradiation, stressing the impact of vascular stability loss, leakage and secondary endothelial dysfunction^29^.

To further investigate those findings, the main goal of this study was to investigate the impact of a 16 Gy simulated SRT procedure on different human retinal target cells in vitro. In nAMD, responsible cell types for retinal morphological changes include RPE, endothelial cells and pericytes. The change in their phenotypical behavior i.e., in cell proliferation, cell viability and cell death after irradiation was investigated.

## Results

### Irradiation does not induce cell death of RPE, hPC and HUVEC

**Figure 1:**
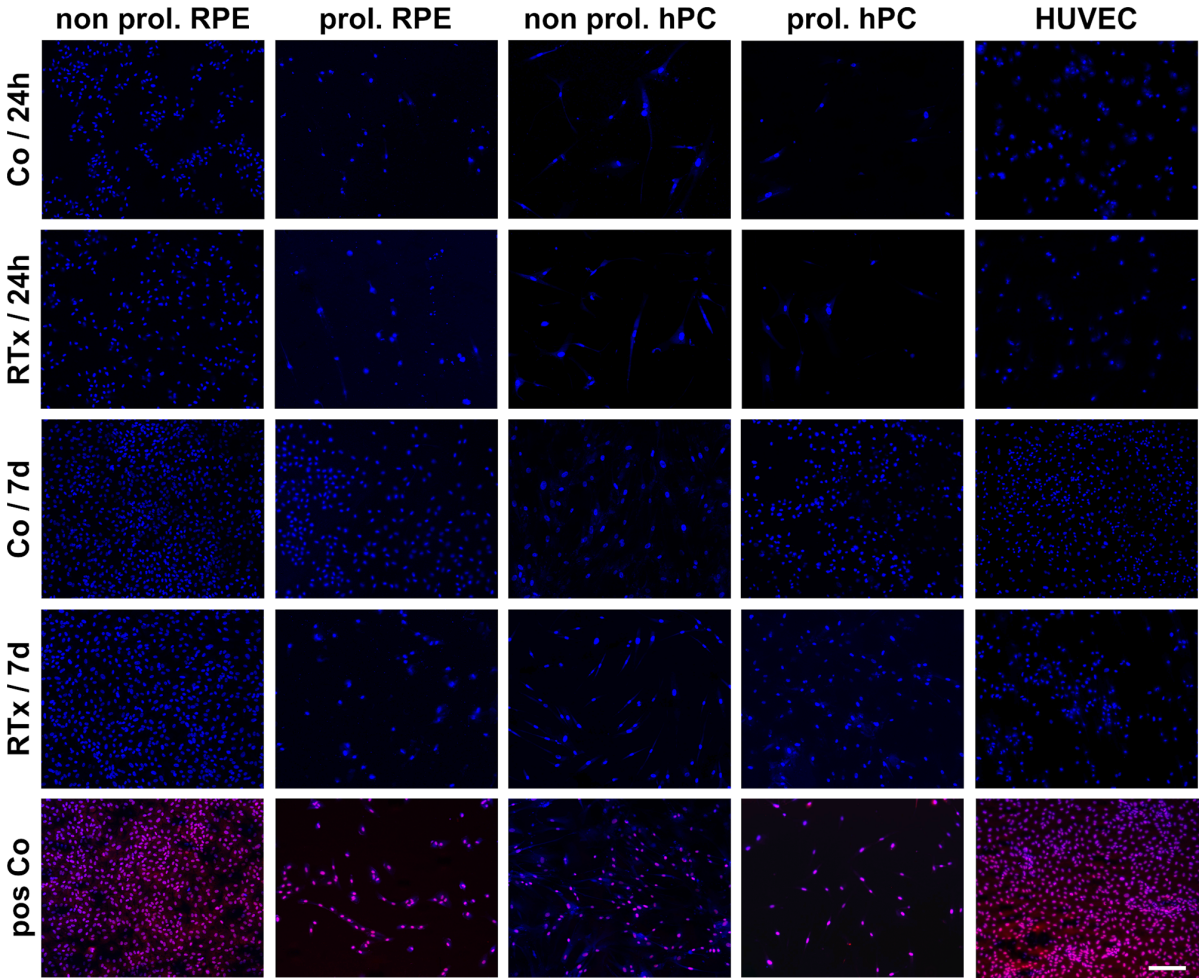
Irradiation does not induce apoptotic cell death of RPE, hPC and HUVEC. Life / Dead staining with Hoechst 33,342 (blue) and propidium iodide (red) was performed 24 h (24 h) and 7 days (7d) after irradiation treatment with 16 Gy (RTx). Magnification bar: 200 µm. Co: untreated control cells; RTx: irradiated cells. Figure was created using Graph Pad Prism 8 (Version 8.4.3.; www.graphpad.com).

To analyze if the applied X-ray radiation induces cell death in the targeted cells, life/dead staining was performed. In untreated controls, only a few cells showed propidium iodide labeling of apoptotic cell nuclei. The number of dead cells in the treated groups was not increased 24 h and 7 d after X-ray exposure when compared to the unirradiated control. (Fig. 1) As expected, after treatment with methanol, a propidium iodide positive staining was observed in almost all cells in each cell line.

### Irradiation reduces cell viability of RPE, hPC and HUVEC

**Figure 2:**
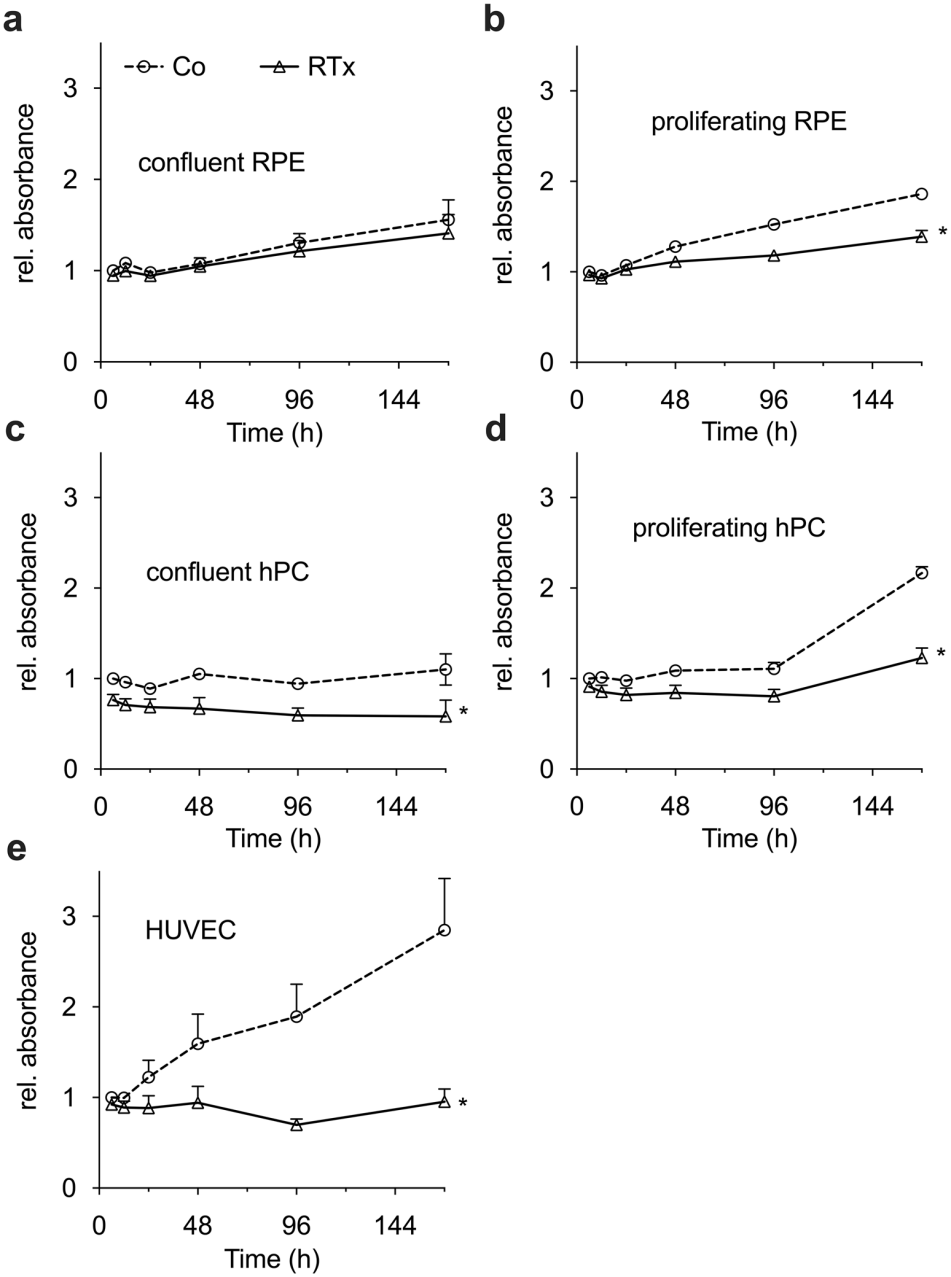
Irradiation reduces cell viability of RPE, hPC and HUVEC. Human RPE, hPC and HUVEC cultured under permissive or not-permissive conditions were irradiated with 16 Gy. XTT cell viability assay was performed at various times after treatment and plotted as relative fold change to the untreated control group 6 h after treatment. *p < 0.05; n = 16 of two independent experiments. Co: untreated control group; RTx: irradiated group; Figure was created using Graph Pad Prism 8 (Version 8.4.3.; www.graphpad.com).

To investigate the effects of 16 Gy SRT on cell viability of human RPE cells, hPC and HUVEC, an XTT assay was conducted. The viability changes of all irradiated cell lines in comparison to untreated controls under permissive and non-permissive conditions over an incubation period of 7 days was compared to the control ‘group’ cell viability at the first measuring point (Fig. 2).

In detail, six hours after irradiation, no statistically significant difference was observed between untreated controls and irradiated cells in all cell lines under permissive or non-permissive conditions. After 7 days, no statistically significant difference in cell viability could be detected between untreated controls and irradiated RPE cells under non-permissive conditions (Fig. 2a). However, a statistically significant difference between untreated controls and irradiated cells could be observed in all three cell lines under permissive conditions (Fig. 2b,d,e) and in hPC under non-permissive conditions (Fig. 2c). Overall, our observations strongly suggest that with the exception of RPE cells under non-permissive conditions, irradiation with 16 Gy inhibits cell viability of the cells.

### Irradiation inhibits cell proliferation

**Figure 3:**
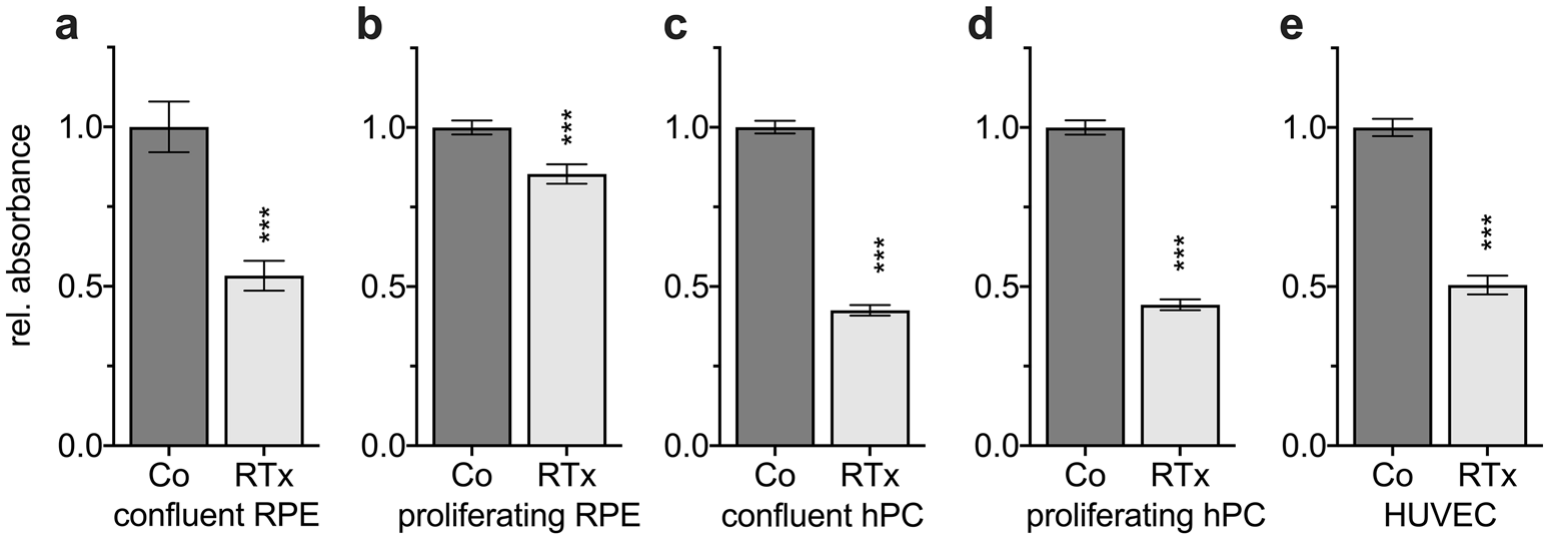
Irradiation inhibits cell proliferation of RPE cells, hPC and HUVEC. Human RPE, hPC and HUVEC cultured under permissive or non-permissive conditions were irradiated with 16 Gy. BrdU proliferation assay was performed 7 d after treatment and plotted as relative fold change to the untreated control group. ***p < 0.001; n = 48 of three independent experiments. Co: untreated control group; RTx: irradiated group; Figure was created using Graph Pad Prism 8 (Version 8.4.3.; www.graphpad.com).

To distinguish between the possible reduction of cell viability and proliferation, a BrdU ELISA was performed. Normalized against their respective unexposed control group, all five cell lines showed a significantly lower proliferation rate at the 7 d endpoint of incubation (Fig. 3). Irradiation inhibited proliferation of RPE cells cultured under permissive conditions by 15% (0.85 ± 0.02; Fig. 3b) and interestingly, the antiproliferative effect of irradiation was stronger in the other cell lines. Non-permissive RPE cells were significantly inhibited in proliferation (0.53 ± 0.03; Fig. 3a) as well as hPC, regardless of whether being cultured under non-permissive (0.42 ± 0.01; Fig. 3c) or permissive conditions (0.45 ± 0.01; Fig. 3d). HUVEC were also significantly inhibited (0.51 ± 0.02; Fig. 3e). Overall, our findings strongly suggest that irradiation inhibits proliferation of human RPE cells, hPC and HUVEC with a smaller effect on activated, permissive RPE cells.

## Discussion

X-rays are a type of radiation, which has sufficient energy to cause ionisation of atoms in biological molecules. This may lead to covalent bond formation within and between macromolecules, potentially including DNA and other cellular components. Especially the damage to the DNA of targeted cells can affect cellular behavior and conveniently, rapidly dividing cells of abnormal morphology are affected more readily^30,31^. On the other side, non-permissive cells are more likely to repair the induced damage and remain structurally intact^32^. In nAMD there is choroidal neovascularization (CNV), which is characterized by abnormal cell proliferation. Radiation is known to preferentially target proliferative cells due to a higher damage susceptibility during DNA synthesis. Provided by this basis, irradiation of patients suffering from nAMD had first been proposed about 30 years ago. Unfortunately, clinical data was less favorable and the application was given up due to poor outcomes^33,34^. The interest in irradiation as an adjuvant therapy was raised again, when a new device was developed and available for commercial use. It delivers low dosage collimated X-ray beams directly to the fovea and therefore avoids irradiation of surrounding healthy tissue as much as possible^35–37^. The device is not in use for a use-alone treatment, due to the state of the art anti-VEGF^38^. Yet, some clinical studies reported that stereotactic radiotherapy might be favorable as an adjuvant treatment for nAMD compared to monotherapy with anti-VEGF agents alone^11,14,39,40^. In addition, research on SRT has recently shown that it may be beneficial for specific forms and characteristics of nAMD (i.e. small CNV lesion)^12,41,42^. Surprisingly however, longer follow-up studies observed a reduced final visual acuity, a higher complication rate and a significant increase in microvascular anomalies when comparing combined SRT and anti-VEGF to anti-VEGF monotherapy^11,16,19^. Unfortunatelly, this clinical data opposed the theoretical expectations, that the apoptotic and anti-proliferative effect on vascular endothelial cells by radiation further inhibits neovascularisation to reach better outcomes compared to anti-VEGF monotherapy^43^. To our knowledge, no experimental study has specifically evaluated the effect of 16 Gy at a cellular level, simulating an IRay-therapy. Therefore, this study treated RPE cells, HUVEC and hPC-PL with low-dose kilovoltage X-ray radiation, thus best simulating this specific treatment mode, in order to figure out the characteristic changes that are induced in healthy cell lines after one single radiation treatment. For technical reasons, we did not use an IRay device, as it does not allow to irradiate cells that are fixed to the cell culture plastic in a horizontal plane.

Our aim was not to provide information on SRT during anti-VEGF treatment, but rather to investigate the influence of a simulated setting of IRay treatment on a cellular level. Based on our investigation, confluent RPE cells were not statistically affected in their viablitity determined by XTT, whereas all other cells seem to be affected in the short-term after low-dose irradiation. Also, in proliferating RPE cells, both -cell viability and proliferation- were statistically significantly reduced after irradiation. Interestingly, in non-proliferating RPE cells, radiation showed only a very low effect on cell viability, but a statistically significant antiproliferative effect. These results are in agreement with previous study findings on RPE cells by confirming the suppressing effect of irradiation on RPE cells’ proliferation and viability without inducing apoptosis^21,23^. Our BrdU assay results indicate an antiproliferative effect on both confluent and proliferating human RPE-cells. While an adequate, clinically satisfying explanation cannot be provided at this stage, we assume that both types of human RPE cells are sensitive to the applied irradiation. Unfortunatelly a direct comparison between the two conditions cannot be drawn from our data, as a different control group was used, one with proliferative and one with confluent cells. Damage to the RPE cells would be an unwanted side effect as it leads to RPE atrophy with consecutive photoreceptor loss. This notion should clinically be further investigated by fundus autofluorescence in order to evaluate RPE changes before treatment and during follow-up.

In vitro, HUVEC as well as hPC-PL—representing vascular cells involved in the process of nAMD—seem to be sensitive to low dose-irradiation. While the life/dead assay did not show an increase in cell death for all irradiated cell lines, the BrdU assay demonstrated a clear inhibition of proliferation at the 7 d endpoint after irradiation. This result might indicate an inhibiting effect of irradiation on neovascular processes in retinal diseases. Furthermore, the XTT assay showed a reduced cell viability of all three cell lines. Interestingly, viability of hPC-PL under non-permissive conditions seemed to remain mainly unchanged over the incubation period of 7 d. On the other hand, the viability of hPC-PL under permissive conditions remained stable over the first 3 d post-irradiation before subsequently rising, showing a short effect of low dose irradiation. Previous studies have shown the effect of low-dose irradiation on HUVEC, indicating a high sensitivity to irradiation^24,26,27^. Structural changes of HUVEC lead to senescence, mechanical changes, dysfunction and apoptosis. Our assays also showed a reaction of HUVEC to the applied radiation, supporting the assumption of inhibiting their vessel forming abilities in nAMD through irradiation. With regard to pericytes, a former study on brain tissue reported severe function loss of pericytes resulting in vascular and endothelial dysfunction and leakage in brain vessels^29^. This fact is in line with our in vitro findings of reduced proliferation and viability, especially under permissive, neovascular-mimicking conditions.

Clinical studies on IRay treatment in nAMD have shown that patients with active leaking lesions, CNV < 4 mm^2^ and increased choroidal thickness demonstrated a better treatment response to the IRay therapy. In addition, a thicker choroid required less injections over the first 12 months after irradiation^12,42,46,47^. The precise targeting of small macular areas < 4 mm^2^ also reduces secondary irradiation of healthy tissue^48,49^. This might explain the positive effect of adjuvant SRT to the anti-VEGF therapy. However, it remains unclear how the healthy retinal and choroidal vessels surrounding active lesions are affected by the SRT. We demonstrated a 50% loss of cell-function of healthy HUVEC and hPC-PL after irradiation rather than an induced apoptosis. In this regard, the increased incidence of microvascular abnormalities after stereotactic radiotherapy in two recent studies may be interpreted as vascular instability and permeability loss of endothelial cells and pericytes^19^. Similar findings that showed radiation induced vessel-necrosis, were observed after irradiation of brain-tumors^29^. As eye and brain are both parts of the central nervous system consisting of neural tissue, one could support a possible similar underlying pathomechanism in retinal and choroidal vessels as in cerebral vessels, though the applied radiation dose is of course much higher in brain tumors.

To gain further insights into these processes and treatment effect, CNV activity, size and volume require a close monitoring of the vascular network of the posterior pole with multimodal imaging, including fluorescein angiography and OCT-angiography before IRay treatment and during follow-up. Furthermore, irradiation of healthy RPE cells at the outline borders of the CNV might lead to a premature macular atrophy and should be monitored regularly by autofluorescence^50^.

In conclusion, this laboratory investigation shows that RPE cells seem not to be as highly affected by the applied low-dose irradiation compared to HUVEC and hPC-PL, especially if in a proliferating phase. As nAMD represents a pathology of complexly and depending interacting cells, the effects of radiation in nAMD patients should be further monitored closely, by applying multimodal imaging for recording of vascular network and macular atrophy.

## Materials and methods

### Ethics approval and consent to participate

For all experimental procedures and tissue harvesting process proper informed consent was obtained. Study approval complied with the Declaration of Helsinki and was provided by the institutional review board of the Department of Ophthalmology, Ludwig-Maximilian University Munich. For the isolation, study and cultivation of postmortem human eye tissue ethical approval was granted by the Ethics committee of the Ludwig-Maximilian University Munich, Germany (Approval ID: 73416; Ethikkommission bei der Medizinischen Fakultät der LMU München, Pettenkoferstr. 8, 80336 München, Germany; Head: Prof. Dr. Eisenmenger). Proper informed consent for the donation and scientific use of the human donor tissue in conformity with the declaration of Helsinki was obtained from all subjects prior to their death by the adult donors themselves or, if subjects were under 18, from a parent or legal guardian. For post-mortem donors proper informed consent was provided from guardian of the next of kin.

### Cell culture

Cells were cultured in cell culture plates (NUNC, Langenselbold, Germany) under standard cell culture conditions (37 °C and 5% carbon dioxide). Cell culture medium was supplemented with 50 IU penicillin/ml and 50 μg streptomycin/ml (Merck Millipore, Burlington, Massachusetts, USA) and replaced every second day. To evaluate the effects of simulated IRay irradiation on cellular proliferation, viability and toxicity, three representative cell lines were used. Human umbilical vein endothelial cells (HUVEC) and human pericytes from placenta (hPC-PL) were obtained as primary, proliferating, freshly isolated cells (PromoCell, Heidelberg, Germany). Either pericyte growth medium or endothelial cell growth medium with supplement growth factor mix and 2% FCS (PromoCell) were used. After primary RPE cell cultures were established through isolation, cells were subcultured and maintained in DMEM (Biochrom, Merck Millipore) supplemented with 10% FCS (Merck Millipore). Subculturing was performed at 80% confluency and all experiments were performed between passages two and five.

### Isolation of primary human RPE cells

Primary RPE cells were isolated from three human donor eye pairs, which were obtained from the institute of forensic medicine, Ludwig-Maximilians-University Munich, Germany and processed within 24 h after death. None of the donors had a known history of eye disease. The isolation of human RPE cells was performed as described previously^51^. In brief, whole eyes were cleansed in 0.9% NaCl solution (B. Braun, Melsungen, Germany), immersed in 5% poly(1-vinyl-2-pyrrolidone)-iodine (B. Braun) and rinsed again in 0.9% NaCl solution. After removal of the anterior segment of the eye for transplantation purposes, the remaining bulbar tissue was transferred to the laboratory. Retinal disease was ruled out by binocular stereomicroscope examination. The neural retina was detached from the RPE layer and the eye cup was washed with a 1 × phosphate-buffered saline (Merck Millipore) / 1 mM EDTA (Sigma, St. Louis, Missouri, USA) for 20 min. A dissociation buffer containing 3 mM l-cysteine (Sigma), 1 mg/ml bovine serum albumin (Biomol, Hamburg, Germany) and 1 U/ml papain (Worthington, Lakewood, New Jersey, USA) was prepared and the eyecup was incubated with the buffer for 23 min at 37 °C. The media was carefully agitated, releasing the RPE into the media and protecting the Bruch’s membrane. Centrifugation followed at 800 rpm for 4 min at room temperature. The RPE pellet was resuspended in DMEM (Merck Millipore) with 20% FCS, seeded and checked for cross contamination using a microscope.

### Treatment

To simulate the impact of SRT on pathological, proliferating cells as well as on physiological, resting cells in nAMD, the highly proliferative RPE and hPC cell lines were studied under serum-free -hereinafter referred as non-permissive- and serum-stimulated -hereinafter referred as permissive- conditions. HUVEC were only examined under proliferating conditions.

For analysis of cell viability and proliferation, cells under non-permissive conditions (3.2 × 10^4^ cells/cm^2^) and cells under permissive conditions (1.6 × 10^4^ cells/cm^2^) were seeded onto a 96-well cell culture plate and incubated for 24 h. For the simulation under non-permissive conditions, the medium containing FCS of the quiescent cell lines was removed and cells were thoroughly washed three times with 1 × phosphate-buffered saline (PBS, Merck Millipore) and exposed to serum-free cell culture medium for another 24 h before irradiation was performed (non-permissive condition). For the simulation of a high proliferative cellular type, all three cell types were kept under standard cell culture conditions in supplemented, serum-containing cell culture medium until irradiation was performed 24 h after seeding and throughout the following incubation period.

For life/dead staining under permissive conditions, cells were grown on round 15 mm microscopy glass coverslips (Thermo Fisher Scientific, Waltham, Massachusets, USA). After attachment over 24 h, cells were irradiated. As positive control, cells were treated with methanol for 10 min.

### SRT protocol

Initially, two plates were prepared as identical twins, plated at the exact same time and cellular density and kept under the exact same conditions until required confluency was reached. For irradiation, both plates were removed from the incubator simultaneously. While one plate was irradiated in an xStrahl RS225 irradiation cabinet (Xstrahl, Camberley, United Kingdom), the other was kept next to the irradiator in the same room at the same environmental conditions.

Complying with the IRay stereotactic low-voltage X-ray irradiation treatment system for age-related macular degeneration, irradiation was performed using low-energy X-ray radiation with converging beams at the cell level of the plates and achieving a radiant energy of 103 keV (200 kV tube voltage, 10 mA amperage)^52^. The SRT for nAMD was mimicked by administering 16 Gy to the cell plates by exposing them to 200 kV and 10 mA for 16 min and 48 s^53^.

### Cell viability

After irradiation and incubation of the cells for the respective time period, a tetrazolium dye reduction assay (XTT, Sigma) was performed to evaluate the change in metabolic activity, reflecting cellular viability. The XTT assay was used as previously described with little modifications^52^. In brief, cells were washed with PBS and 1 ml of 0.08 mg/ml XTT solution in clear culture medium without phenol red was added to each well and incubated at 37 °C for 30 min. After the removal of the XTT solution, 1 ml of DMSO was added for formazan crystal solubilization. Absorption was measured at 450 nm using a spectrophotometer (SpectraMax 190, Molecular Devices, San Jose, California, USA). XTT substrate metabolism was measured at various times between 6 and 168 h after irradiation and plotted as relative fold change to the untreated control after 6 h.

### Proliferation

To determine cell proliferation after irradiation treatment, a 5-bromo-2′-deoxyuridine (BrdU) ELISA (Merck Millipore) was performed as described previously in accordance to the manufacturer’s recommendations^53^. Immediately after irradiation and before incubation for 7 d, the BrdU labeling solution was added. After incubation, cells were fixed and anti-BrdU antibodies were added. After 2 h, the antibody solution was replaced by the substrate developing solution. Reaction products were quantified by absorbance measurement at a wavelength of 450 nm/690 nm on the SpectraMax 190 (Molecular Devices).

### Life/dead assay

After incubation for 24 h and 7 d in their respective cell culture medium after irradiation the slides were washed three times with 1 × PBS before incubation with 1 µg/ml Hoechst 33342 and 2 µg/ml propidium iodide (both from Invitrogen, Carlsbad, CA) in cell culture medium for 15 min following additional three washings with 1 × PBS. After fixation of the cells with 4% paraformaldehyde for 10 min, the cells were washed again three times with 1 × PBS and mounted with antifade mounting medium (Vector Laboratories, Burlingame, California, USA). Staining was analyzed on an inverted Axio Observer 7 with Apotome2 module (Zeiss, Oberkochen, Germany) and documented by using the ZEN software (Zeiss).

### Statistical analysis

EXCEL 365 version 16 (Microsoft, Redmond, WA), SPSS version 24 (IBM, Armonk, NY) and GraphPad PRISM 8 (Version 8.4.3, GraphPad Software, Inc., San Diego, CA) was used for calculations, analysis and presentation of the data. In accordance with the necessary comparison a 1-way ANOVA with a least significant difference (LSD) post-hoc test was performed for data meeting the assumption of homogeneity of variances and a Games Howell post hoc for data not meeting the criteria. A *p-*value < 0.05 was regarded statistically significant. P values < 0.05 were marked with * in graphs, p < 0.01 with ** and p < 0.001 with ***. Error bars represent the standard error of the mean.
